# Twelve-Week Gait Retraining Reduced Patellofemoral Joint Stress during Running in Male Recreational Runners

**DOI:** 10.1155/2020/9723563

**Published:** 2020-03-20

**Authors:** Baofeng Wang, Yang Yang, Xini Zhang, Junqing Wang, Liqin Deng, Weijie Fu

**Affiliations:** ^1^School of Kinesiology, Shanghai University of Sport, Shanghai 200438, China; ^2^Key Laboratory of Exercise and Health Sciences of Ministry of Education, Shanghai University of Sport, Shanghai 200438, China

## Abstract

**Purpose:**

To explore the changes in knee sagittal angle and moment and patellofemoral joint (PFJ) force and stress before and after 12-week gait retraining.

**Methods:**

A total of 30 healthy male recreational runners were randomized into a control group (*n* = 15) who ran in their original strike pattern using minimalist shoes or experimental group (*n* = 15) who ran in a forefoot strike pattern using minimalist shoes during the 12-week gait retraining. The kinematic and kinetic data of the dominant leg of the participants during the 12 km/h running were collected by 3D motion capture systems and 3D force platforms. Besides, the biomechanical property of the PFJ was calculated on the basis of the joint force model and the regression equation of the contact area.

**Results:**

After the 12-week gait retraining, 78% of the rearfoot strikers turned into forefoot strikers. Peak knee extension moment and peak PFJ stress decreased by 13.8% and 13.3% without altering the running speed, respectively. Meanwhile, no changes in maximum knee flexion angle/extension moment and PFJ force/stress were observed for the control group.

**Conclusion:**

The 12-week gait retraining effectively reduced the PFJ stress, thereby providing a potential means of reducing the risk of patellofemoral pain syndrome while running.

## 1. Introduction

Running is a popular and prevalent way of exercising [1, 2]. In the United States alone, almost 60 million people participated in jogging, running, and trail running in 2017 [3]. Running-related injuries (RRI) have attracted the attention of researchers because of the increasing number of runners. Previous studies reported that RRI accounted for 40% of injuries caused by exercise [4]. Among RRI, those related to the knee had the highest ratio at 28%; in particular, patellofemoral joint pain accounted for the highest proportion (17%) of the specific pathologies of injury [5].

To date, high patellofemoral joint stress, overuse, trauma, decreased elasticity in quadriceps femoris, limited motion of the patella, and contracture of the patellofemoral lateral supporting band are regarded as the main causes of patellofemoral pain syndrome (PFPS) [6, 7]. Nonsurgery curative treatments are carried out through the strength training of the quadriceps, medial oblique femoris, and gluteal muscle to correct the movement trajectory of the patella [8, 9]. However, the aforementioned treatments are usually applied only after the occurrence of PFPS. PFPS caused by running is also mainly triggered by the interaction of increased patellofemoral joint stress and weak strength of the lower extremity muscles. Thus, the important factor of developing stress on the patellofemoral joint may be neglected when only muscle strength is increased.

Runners can be divided into rearfoot strikers (RFS), midfoot strikers, and forefoot strikers (FFS) based on their strike pattern [10]. A total of 75% of the runners who were used to wearing cushioned shoes correspond to rearfoot strikers [11]. In FFS, the ankle was more plantarflexed at initial contact than in RFS. The foot went through greater dorsiflexion range of motion during stance in FFS during running because of the increased plantarflexion [12]. Such change allowed shock absorption by the muscles and ligaments of the foot, which decreased loading rates and work at the knee compared with running with a rearfoot strike pattern [13–15]. Warne et al. showed that a six-week combination program of gait retraining and minimalist shoes could reduce the loading rate and peak impact force by transforming the pattern of RFS to a nonrearfoot strike pattern [16, 17]. Female natural FFS had lower extension moment, patellofemoral joint contact force, and patellofemoral stress than those in the RFS group [18]. Researchers transversely compared the biomechanical data between these two strike patterns [18] and the acute changes in their longitudinal posture to analyze their differences [19]. Moreover, most studies of gait retraining have targeted runners with patellofemoral pain [20], ignoring the different running patterns such as greater hip adduction and internal rotation between runners with patellofemoral pain and healthy runners [21]. Meanwhile, previous studies have reported that persons with patellofemoral pain may have an abnormal joint structure (i.e., patella malalignment and patella alta) that could influence joint contact mechanics [22]. The differences in kinematics and joint structure between runners with and without patellofemoral joint pain may cause runners to adapt to gait retraining differently. Only one study done by Dos Santos et al. showed that healthy runners with the forefoot strike pattern exhibited lower patellofemoral joint stress compared with the rearfoot strike pattern [19]. However, habitual runners acutely translated to forefoot strikers by verbal instructions from the examiner. This period should not provide the participants with a process of adaptation potential changes in strike patterns and related loads. Therefore, fully understanding the biomechanical effects of patellofemoral stress in healthy runners by gait retraining is necessary.

Based on the above observation, this study was aimed at exploring the changes in knee sagittal angle, sagittal moment, and patellofemoral joint contact force (PFCF) and stress (PFS) before and after 12-week gait retraining. Thus, preventive measures of PFPS are expected to reduce injury rates. We hypothesized that the participants of the EG converted to the forefoot strike pattern with a lower foot strike angle after the 12-week gait retraining. Besides, runners would exhibit lower patellofemoral joint contact force and stress as a consequence of the 12-week gait retraining.

## 2. Methods

### 2.1. Participants

An a priori power analysis was conducted for expected outcomes with a type I error probability of 0.05 and a power of 0.8. This analysis indicated that *n* = 16 (total sample size) would provide a statistical power of approximately 80% (G∗Power v3.1.9.4). To utilize a control group and to allow attrition from the study, 30 male participants were recruited and divided into experimental (EG) and control groups (CG) (i.e., 15 participants for each group) using a random order. The type of randomization was designed for simple randomization. Randomization was performed by one researcher. The function “Rand ()” was used to generate a random number between each participant that corresponds to one “0–1” (e.g., 0.60621385), and the participants were divided into experimental and control groups in an ascending order, with 15 people in each group. No stratification or blocking factor was used. The inclusion criteria are presented as follows: (1) recreational runners who are inclined to rearfoot strike pattern and wearing cushioned shoes; (2) a weekly running distance of over 20 km in the four recent weeks and has the ability to maintain this distance for the next 3 months; and (3) should be free from lower extremity injuries within 3 months. This study, with detailed guidelines for participants' safety and experimental protocols, was approved by the Institutional Review Board of the Shanghai University of Sport (No. 2017007). The study was conducted in accordance with the declaration of Helsinki. Specifically, all procedures and potential hazards were clarified to the participants in nontechnical terms, and informed consent was signed prior to the tests. All participants were with full knowledge of test procedures and requirements.

### 2.2. Experimental Design

We designed a parallel randomized control group to compare the effects of 12-week intervention in the experiment group of participants assigned to gait retraining with the effects in the control group. Participants were classified into experimental and control groups randomly with an allocation ratio of 1 : 1 according to computer-generated random numbers. The primary outcomes corresponded to changes from baseline in the patellofemoral joint stress and contact force. Secondary outcomes included the changes in knee extension moment, knee flexion angle, and foot strike angle.

### 2.3. Instrumentations

**Figure 1:**
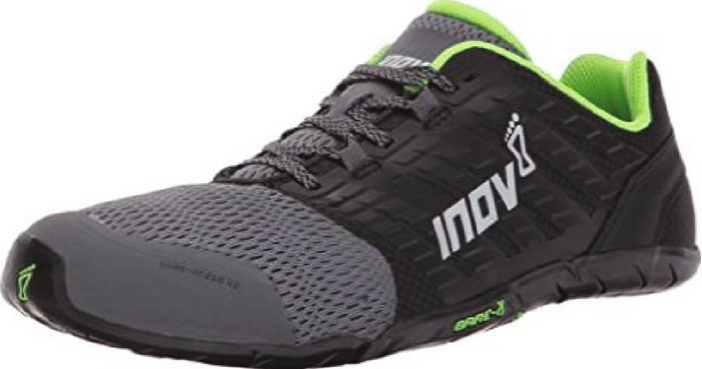
INOV-8 Bare-XF 210 V2.

Two 90 cm × 60 cm × 10 cm Kistler 3D force platforms (9287B, Kistler Corporation, Switzerland) were used to collect ground reaction force (GRF) data at a sampling rate of 1000 Hz. Forty infrared retroreflective markers (diameter: 14.0 mm) were attached bilaterally to both lower extremities to define hip, knee, and ankle joints according to the plug-in gait marker set [23]. A 10-camera infrared 3D motion capture system (Vicon T40, Oxford Metrics, UK) was utilized to collect the trajectory markers at a sampling rate of 100 Hz. Running speed during the experiment was controlled by a Witty-Manual grating timing system (Microgate, Italy). The sole thickness and average weight of INOV-8 Bare-XF 210 V2 minimalist shoes (Figure 1), which did not contain any cushioning material and heel-toe drop, were 3 mm and 227 g, respectively. The size of the experimental shoes ranged from EUR 41 to 43 based on the foot size of the participants. A Podoon© pressure-sensitive intelligent shoepad, in which three flexible thin pressure sensors were inserted and could be coordinated with the Podoon© app, was used to monitor foot strike patterns during training.

### 2.4. Experimental Procedure

Basic information of the participants and informed consent forms were filled in, physical fitness was tested, and the experimental procedure was explained before training. The participants were required to wear experimental vests, shorts, and socks before the running experiment. Then, the participants underwent a 5-minute warmup at a speed of 12 km/h on a treadmill followed by 5 minutes of rest to enable participants to change into their minimalist shoes. 36 markers were attached to the bony landmarks of the body based on the plug-in-gait marker set [24]. Before the formal testing, the static models of the participants were captured. The participants ran overground at a speed of 12 km/h (±5%) using self-selected strike patterns (i.e., the grating timing system was used to control speed). The trajectory of markers and GRF data were collected simultaneously. Three successful running trials were collected for each participant with the dominant leg stepped on the force platform (the dominant leg was determined by kicking a ball [25]).

### 2.5. Retraining Intervention

For the EG, the participants were required to wear minimalist shoes when executing retraining to run at a self-selected speed with moderate intensity. Forefoot strike pattern was required; i.e., the participants should use the metatarsal ball of the forefoot to strike the ground first, in which the foot was placed below the hip during landing [26].

For the CG, the participants were also required to wear minimalist shoes and maintain their original strike pattern when training at a self-selected speed with moderate intensity. However, no other instructions were provided.

The intervention period lasted for 12 weeks. Time-incremental training sessions were held three times a week. Each training session lasted for 5–48 minutes across the 12 weeks (Table 1). The participants were allowed to wear habitual running shoes when out of training. During training sessions, the two groups were prevented from interacting with one another.

Matches were banned during the entire intervention period. The training plan only partly substituted for the duration of running, and the overall weekly running distance remained constant. Moreover, participants were instructed to enhance the strength and function training of their foot and lower extremity muscles to adapt to potential changes in strike patterns and related loads (Table 2) [27].

The participants were required to record the training conditions in their retraining diaries. The CG needed to record the starting time, ending time, site, and injury conditions during training. The EG needed to record the distances of running with the forefoot strike pattern in addition to the abovementioned recordings. Meanwhile, the participants of each group are required to record their own physical conditions. When the participants experience discomfort or injury, the researchers will determine whether the participants can still continue to train according to their conditions. Researchers should be informed about the specific site and starting time of training during random checks. The cloud data of intelligent insole and retraining diaries were compared during the intervention period. The experimenter would inform the participants who do not meet the requirements or with data mismatch in the cloud through telephone or online. Participants who are discontinued for more than a week will be excluded.

### 2.6. Data Processing

In this study, stance phase was identified from touchdown to toe-off. Marker trajectories were filtered with a cutoff frequency of 7 Hz via Visual 3D gait analysis software (v5, C-Motion, Inc., Germantown, MD, USA). Excel 2016 was used to extract characteristic values.

Foot strike pattern was identified through the curve of the vertical GRF (vGRF) during overground running [13]. Meanwhile, the foot strike angle was calculated by taking the angle of the foot at touchdown while running and subtracting the angle of the foot while standing.

In the current study, the patellofemoral stress under a dynamic condition was calculated via biomechanical modeling. Generally, a calculation model of a patellofemoral contact force [28] regression equation of the contact area between the patella and the femur [29] was applied. PFS was calculated on the basis of the abovementioned studies. The details of the cited model are presented as follows.

**Figure 2:**
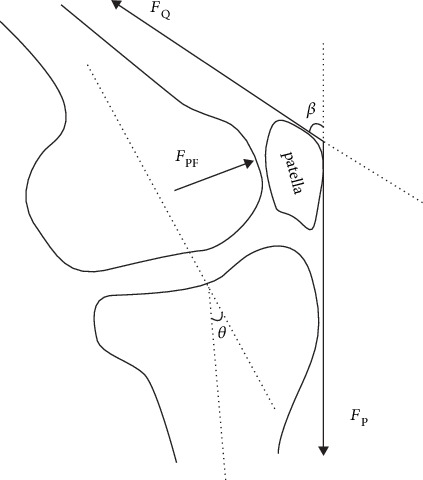
Free-body diagram of the patellofemoral joint and definition of angle.

Quadriceps force was calculated using the following equation:

(1) $$\begin{array}{c} F_{\text{Q}}\;\left(\theta_{\text{i}}\right)=M_{\text{EXT}}\left(\theta_{\text{i}}\right)/L_{\text{A}}\;\left(\theta_{\text{i}}\right), \end{array}$$

where *F*_Q_ is the quadriceps force (N), *M*_EXT_ is the extension moment generated at the knee by ground reaction force acting on the foot (N m) [30], and *θ*_i_ is the knee sagittal angle [31] (Figure 2). *L*_A_ (m) is the effective arm of force of quadriceps, which is a function of the knee sagittal angle.

(2) $$\begin{array}{c} L_{\text{A}}=\left\{\begin{array}{c} 0.036\theta_{\text{i}}+3.0\left(0^{{}^{\circ}}\leq \theta_{\text{i}}<30^{{}^{\circ}}\right),\\ -0.043\theta_{\text{i}}+5.4\left(30^{{}^{\circ}}\leq \theta_{\text{i}}<60^{{}^{\circ}}\right),\\ -0.027\theta_{\text{i}}+4.3\left(60^{{}^{\circ}}\leq \theta_{\text{i}}<90^{{}^{\circ}}\right),\\ 2.0\left(90^{{}^{\circ}}\leq \theta_{\text{i}}\right). \end{array}\right. \end{array}$$

Patellofemoral joint contact force was calculated as follows:

(3) $$\begin{array}{c} F_{\text{PF}}=2F_{\text{Q}}\sin\;\left(\frac{\beta }{2}\right), \end{array}$$

where *β* = 30.46 + 0.53 · *θ*_i_, *F*_PF_ (N) is PFCF, and *β* (°) is the angle of the quadriceps line and patellar ligament [28] (Figure 2).

Patellofemoral stress was calculated as follows:

(4) $$\begin{array}{c} P_{\text{PFS}}=F_{\text{PF}}/S_{\text{PFCA}}\;\left(\theta_{\text{i}}\right), \end{array}$$

where *P*_PFS_ is the patellofemoral joint stress. The contact area (mm^2^) between the patellar and the femur is a function of the knee sagittal angle [29], which is expressed as follows:

(5) $$\begin{array}{c} S_{\text{PFCA}}=0.0781\times {\theta_{\text{i}}}^{2}+0.06763\times \theta_{\text{i}}+151.75, \end{array}$$

where *S*_PFCA_ represents the contact area between the patellar and femur.

### 2.7. Statistics

The basic information of the participants was tested using the *t*-test of paired samples. A two-way repeated measures ANOVA was used to determine the effects of the 12-week gait retraining on the dependent variables (i.e., knee sagittal moment and angle and patellofemoral joint contact force and stress) (SPSS 21.0). For the interaction parameters, dependent and independent *t*-tests were conducted for interclass and intergroup data, respectively. The significance level was set at 0.05.

## 3. Results

### 3.1. Dropout Rate

Overall, 17 participants (experimental group: *n* = 9; control group: *n* = 8) completed the 12-week gait retraining protocol and had a second visit to the laboratory for posttraining tests (Table 3). In detail, a participant, who was an FFS runner in a test prior to training, was excluded. In the processing of intervention, two participants quit because of injuries caused by nontraining-related incident, i.e., carelessly taking the stairs. Two other participants were excluded because of mismatch in cloud data and diaries without providing reliable evidence, such as apps or intelligent watch data. The cloud data of Podoon© were unable to observe the mismatch, and no correspondence was obtained for the three individuals. Five people who quit training after more than a week were also excluded. In addition, no participants reported that the training intensity/volume was too high/much to follow. More importantly, in the experimental group, 7 of out 9 participants transformed into forefoot strike with a rate of 78%.

### 3.2. Foot Strike Angle

**Figure 3:**
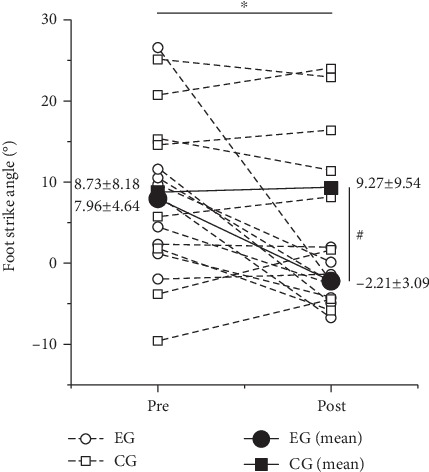
Effects of the 12-week gait retraining on the foot strike angle. ^∗^Significant difference from pre- to posttests in the EG group; ^#^significant difference between groups at time point, *p* < 0.05.

No significant difference was observed in the foot strike angle between the EG and CG at baseline (*p* = 0.126). The main significant effect of time was observed on the foot strike angle (*p* = 0.026). The foot strike angle of the EG group decreased by 10.2° after training (*p* = 0.015); however, no difference was noted in the CG (*p* = 0.753). The foot strike angle of the EG significant differs from that of the CG group in the posttest (*p* = 0.017) (Figure 3).

### 3.3. Knee Sagittal Angle and Moment

**Figure 4:**
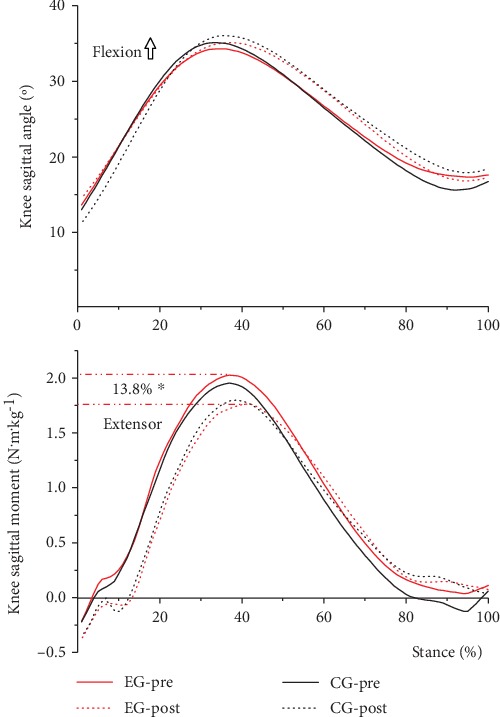
Effects of the 12-week gait retraining on the knee flexion angle (upper) and knee extension moment (lower) (mean values from all participants). Asterisk (^∗^) denotes that postintervention is significantly different from preintervention.

After the 12-week gait retraining, in the EG, the peak knee extension moment significantly decreased by 13.8% (*p* = 0.018) (Figure 4), whereas changes were not observed for the maximum knee flexion angle during the stance phase. Meanwhile, no changes were observed for the maximum knee flexion angle and peak knee extension moment from the CG (Table 4). No between-group and interaction effects were observed for the maximum knee sagittal angle and moment (Table 5).

### 3.4. Patellofemoral Joint Contact Force and Stress

**Figure 5:**
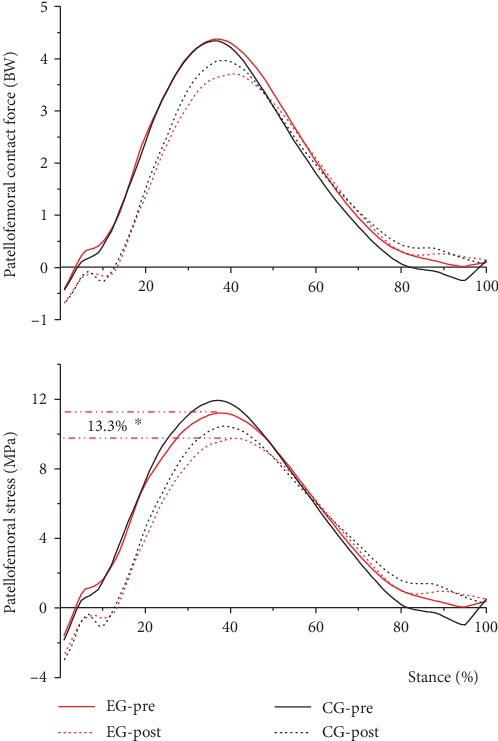
Effects of the 12-week gait retraining on patellofemoral joint contact force (upper) and stress (lower) (mean values from all participants). Asterisk (^∗^) denotes post-intervention significantly different from pre-intervention.

After the 12-week gait retraining, in the EG, a significant decrease of 13.3% was found in peak patellofemoral joint stress (*p* = 0.018) (Figure 5), whereas peak patellofemoral joint contact force remained the same. Meanwhile, no significant changes were observed for peak patellofemoral joint force and stress in the CG after the 12-week gait retraining (Table 4). No significant effect was observed for peak patellofemoral joint contact force and stress between the EG and CG before and after the 12-week gait retraining. In addition, no significant time × group interaction was noted on the peak patellofemoral joint contact force and stress (Table 5).

## 4. Discussion

This study was mainly aimed at exploring the effects of different strike patterns on the mechanism of the patellofemoral joint to provide an effective means of preventing PFPS through the 12-week gait retraining. Results showed that through gait transition and use of minimalist shoes for 12 weeks, the peak knee extension moment and peak patellofemoral joint stress decreased significantly, whereas no significant difference was observed in the CG, which only used minimalist shoes.

A total of 7 out of 9 individuals changed to FFS after the 12-week gait retraining with a rate of 78%. Therefore, the 12-week gait retraining is sufficient for runners to learn gait transition and convert strike pattern. In this research, two participants dropped out because of injuries caused by nontraining-related incident (one person for each group). A total of 2 out of 15 individuals (13%) obtained injury due to training in the study of McCarthy et al. [32]. Furthermore, Warne et al. [27] showed that 2 of 14 EG participants (14%) suffered from hamstring and gastrocnemius strain, and the training time of 7 individuals was reduced due to reported calf pain. The condition of minimalist shoes for this study was also applied in McCarthy et al.'s study. Our study differed because although the participants were informed that pain might be caused by a long-step length and continuous use of rearfoot strike while wearing minimalist shoes, no specific plan was made for participants to actively change their running posture. In Warne's study, the training time was increased to 40 minutes only within 6 weeks. However, in the current retraining protocol, the training volume was increased to 40 minutes in the 8^th^ week; Afterward, a period of 2 minutes was added per week for the last 4 weeks of the research. Injuries and pain did not occur in this research because of the active changing of strike pattern, long intervention time, and low increase rate of training time, suggesting that the proposed intervention is safer for strike pattern transition compared with those of previous studies.

For the mechanical property of the patellofemoral joint, a significant decrease of 13.3% was observed in peak PFS in the EG. Similarly, Kulmala et al. [18] showed that the peak PFS of FFS decreased by 15% compared with that of RFS. In the EG, the peak knee extension moment was significantly reduced by 13.8% after the 12-week gait retraining. Lower peak knee extension moment was also found among FFS compared with RFS in other studies [19, 29, 33]. The abovementioned changes in the EG in the present study were not observed in the CG. Thus, RFS who were trained to be FFS by implementing active changes in landing strategy could decrease extension moment and patellofemoral stress at a constant speed (12 km/h ± 5%). PFS is calculated as PFCF divided by *S*_PFCF_, and the knee sagittal angle is the only variable in the *S*_PFCF_ function. No significant changes in the maximum knee flexion angle were observed before and after retraining. Thus, no statistical difference was determined in *S*_PFCF_. Similarly, changes in the *β* angle and LA were insignificant, suggesting that PFS decreased mainly because of the reduction in the peak knee extension moment. Liao et al. focused on the difference in patellofemoral joint stress between participants with or without patellofemoral joint pain based on the finite element model and found that the peak PFS of runners with pain was larger than that of runners without pain and knee moment was regarded as a predictive factor [34]. Previous research suggests that FFS had lower vGRF than RFS [35], FFS could have a larger ROM of ankle [33], and the mechanical properties of the Achilles tendon can be strengthened by forefoot strike. The improvement in the loading ability of the Achilles tendon is beneficial to the calf to play a greater role in the impact phase. Lower vGRF and increased loads on the ankle using forefoot strike may cause lower peak knee extension moment [12].

Patellofemoral pain syndrome is the most common overuse injuries among runners [36]. Long-term patellofemoral joint pain can increase the probability of patellofemoral arthritis [37]. Studies showed that an increased PFS is a triggering factor of patellofemoral pain. A high PFS can lead to cartilage degeneration, which causes patellofemoral pain syndrome. Overall, the present study found that running gait retraining and wearing minimalist shoes enable runners to decrease peak PFS, and cases of injuries did not occur because of training. Therefore, the gait retraining scheme in this study was effective in preventing patellofemoral joint pain caused by large PFS while running and provided a potential means of lessening patellofemoral joint pain in runners.

Although the gait retraining scheme was effective and safe, certain limitations should be considered. The number of samples finally recruited was relatively small because of participant dropout. In the future research on recreational runners, we should focus on the training control of the participants because of work travel and other reasons, which are difficult but useful for sample preservation. In addition, intention-to-treat analysis was not conducted due to lack of second test data. Moreover, the effects caused by gait retraining in female recreational runners are unknown. Thus, female recreational runners should be recruited in future studies.

## 5. Conclusion

The sole use of minimalist shoes cannot influence the mechanical property of the patellofemoral joint. However, the 12-week gait retraining with minimalist shoes changed RFS to FFS and reduced knee extension moment and PFS without altering the running speed. Thus, the 12-week gait retraining intervention applied in this study can effectively decrease patellofemoral joint loads and provide a potential means of reducing the risk of patellofemoral pain syndrome caused by large PFS while running.

## Figures and Tables

**Table 1:** A 12-week gait retraining protocol.

Week	1	2	3	4	5	6	7	8	9	10	11	12
Time (min)	5	10	15	20	25	30	35	40	42	44	46	48
Frequency (times/week)	3	3	3	3	3	3	3	3	3	3	3	3

**Table 2:** A 12-week foot and ankle exercise program.

Week	1	2	3	4	5	6
Double leg heel raises (level surface)	3 × 20	3 × 20	3 × 20	3 × 20	3 × 20	N/A
Double leg heel raises (on step)	N/A	3 × 20	3 × 20	3 × 20	3 × 20	N/A
Single leg heel raise (level surface)	N/A	N/A	3 × 10	3 × 15	3 × 20	3 × 20
Towel curls	3 × 20	3 × 30	3 × 30	3 × 30	3 × 30	3 × 30
Toe spread and toe squeeze	3 × 20	3 × 25	3 × 30	3 × 30	3 × 30	3 × 30
Doming	3 × 20	3 × 25	3 × 30	3 × 35	3 × 40	3 × 40

**Table 3:** Mean ± SD data for basic information of participants (*n* = 17).

Groups	Age (years)	Height (m)	Weight (kg)	Weekly volume (m)
Experimental group (**n** = 9)	32.4 ± 6.1	1.75 ± 0.05	70.2 ± 6.0	28300 ± 11100
Control group (**n** = 8)	27.6 ± 5.2	1.74 ± 0.07	75.4 ± 11.6	26800 ± 10600
**t**-test	**p** = 0.104	**p** = 0.773	**p** = 0.262	**p** = 0.787

**Table 4:** Mean ± SD data for the maximum knee flexion angle, peak knee extension moment, and patellofemoral joint contact force (PFCF) and stress (PFS) before and after the 12-week gait retraining.

Variates	Experimental group (**n** = 9)					Control group (**n** = 8)				
	Pre-	Post-	Mean difference (CI 95%)	**p** value	Effect size	Pre-	Post-	Mean difference (CI 95%)	**p** value	Effect size
Peak knee extension moment (N m kg^−1^)	2.1 ± 0.4	1.8 ± 0.3	0.28 (0.09~0.47)	0.018^∗^	0.36	2.0 ± 0.6	1.9 ± 0.6	0.11 (-0.07~0.27)	0.242	0.08
Maximum knee flexion angle (°)	34.4 ± 2.3	35.4 ± 4.8	-0.86 (-3.66~1.96)	0.569	0.14	35.4 ± 2.9	36.4 ± 4.8	-1.06 (-4.40~2.30)	0.529	0.13
Peak PFCF (BW)	4.5 ± 1.1	4.0 ± 0.9	0.51 (-0.03~1.03)	0.101	0.24	4.4 ± 1.6	4.2 ± 1.7	0.26 (-0.32~0.84)	0.359	0.08
Peak PFS (MPa)	11.6 ± 2.92	10.1 ± 2.2	1.05 (0.55~2.57)	0.017^∗^	0.29	12.1 ± 4.0	10.9 ± 3.2	1.23 (-0.12~2.58)	0.098	0.17

Notes: asterisk (^∗^) denotes significant differences between pre- and postintervention within the group (*p* < 0.05).

**Table 5:** Mean difference data, 95% confidence intervals, *p* value, effect sizes, and power for time, group, and interaction effect.

Variables	**F** test	Mean difference (95% confidence intervals)	**p** value	Effect size	Power
Peak knee extension moment (N m kg^−1^)	Time	0.19 (0.08~0.32)	0.008	0.20	0.61
	Group	0.05 (-0.19~0.27)	0.845	0.05	0.06
	Time × group		0.177	0.17	0.32

Maximum knee flexion angle (°)	Time	0.12 (-2.95~1.05)	0.386	0.12	0.27
	Group	-0.95 (-2.75~0.85)	0.542	0.12	0.09
	Time × group		0.925	0	0.05

Peak PFCF (BW)	Time	0.36 0~0.74	0.066	0.13	0.31
	Group	-0.15 (-0.81~0.53)	0.515	0.05	0.06
	Time × group		0.593	0.19	0.40

Peak PFS (MPa)	Time	1.4 (0.64~2.16)	0.004	0.23	0.73
	Group	-0.64 (-2.34~1.06)	0.668	0.10	0.08
	Time × group		0.698	0.32	0.86

## Data Availability

The table and figure data used to support the findings of this study are included within the article.
